# Laser-ablative aqueous synthesis and characterization of elemental boron nanoparticles for biomedical applications

**DOI:** 10.1038/s41598-022-13066-8

**Published:** 2022-06-01

**Authors:** Andrei I. Pastukhov, Iaroslav B. Belyaev, Julia C. Bulmahn, Ivan V. Zelepukin, Anton A. Popov, Irina N. Zavestovskaya, Sergei M. Klimentov, Sergey M. Deyev, Paras N. Prasad, Andrei V. Kabashin

**Affiliations:** 1 LP3, CNRS, Aix Marseille University, Campus de Luminy, Case 917, 13288 Marseille, France; 2 Institute of Engineering Physics for Biomedicine (PhysBio), MEPHI, Moscow, Russia 115409; 3 Russian Academy of Sciences, 16/10 Miklukho-Maklaya St, Moscow, Russia 117997; 4 Department of Chemistry, The Institute for Lasers, Photonics, and Biophotonics, University at Buffalo, The State University of New York, Buffalo, NY 14260 USA; 5 P. N. Lebedev Physical Institute of the Russian Academy of Science, Leninskiy Pr. 53, Moscow, Russia 119991

**Keywords:** Nanoscience and technology, Nanomedicine, Nanotechnology in cancer, Materials science, Nanoscale materials, Nanoparticles, Optics and photonics, Applied optics, Laser material processing

## Abstract

Boron-based nano-formulations look very promising for biomedical applications, including photo- and boron neutron capture therapies, but the fabrication of non-toxic water-dispersible boron nanoparticles (NPs), which contain the highest boron atom concentration, is difficult using currently available chemical and plasma synthesis methods. Here, we demonstrate purely aqueous synthesis of clean boron NPs by methods of femtosecond laser ablation from a solid boron target in water, thus free of any toxic organic solvents, and characterize their properties. We show that despite highly oxidizing water ambience, the laser-ablative synthesis process follows an unusual scenario leading to the formation of boron NPs together with boric acid (H_3_BO_3_) as an oxidation by-product coating the nanoparticles, which acts to stabilize the elemental boron NPs dispersion. We then demonstrate the purification of boron NPs from residual boric acid in deionized water, followed by their coating with polyethylene glycol to improve colloidal stability and biocompatibility. It was found that the formed NPs have a spherical shape with averaged size of about 37 nm, and are composed of elemental boron in mostly amorphous phase with the presence of certain crystalline fraction. The synthesized NPs demonstrate low toxicity and exhibit strong absorption in the NIR window of relative tissue transparency, promising their use in photoacoustic imaging and phototherapy, in addition to their promise for neutron capture therapy. This combined potential ability of generating imaging and therapy functionalities makes laser-synthesized B NPs a very promising multifunctional agent for biomedical applications.

## Introduction

Exhibiting a series of unique properties, boron and its compounds (boron nitride, boron carbide, e.g.) are widely employed in a variety of applications, including microelectronics^1^, quantum dots synthesis^2^, combustion catalysis^3^, photoelectrochemical hydrogen and oxygen evolution reactions^4^. Biomedicine is also considered as one of potential beneficiaries of boron-based compounds. In particular, boron isotope ^10^B (present in naturally abundant boron) has a high cross section of neutron capture leading to the generation of α particles, which can destroy DNAs of rapidly proliferating cancer cells in order to treat tumors^5,6^. Some boron-containing compounds, including disodium mercaptoundecahydrododecaborate (BSH) and p-boro-phenylalanine (BPA)^5,7^, have already been explored in boron neutron capture therapy (BNCT) and demonstrated a promising therapeutic effect. However, since the eradication of tumor cells requires high concentration of boron-10 isotope (this concentration is estimated to be about 10^9^ atoms^7^ per cell or 20…35 µg/g^8^), heavy doses of molecular boron compounds such as BSH and BPA should be applied to obtain a significant therapeutic effect, which leads to toxicity issues.

Such a bottleneck can be overcome by employing approaches of nanotechnology, which have already proved their efficiency in a variety of fields, including photovoltaics^9,10^, catalysis^11–13^, biomedicine^14,15^. Indeed, the use of boron-based nanoparticles (NPs) instead of molecular agents can offer much larger number of boron atoms and thus enhance the therapeutic outcome. Here, NPs of elemental boron look especially promising as they contain the maximal concentration of boron. Moreover, when NPs are coated by polymers such as polyethylene glycol (PEG), they can have a prolonged circulation in the blood stream due to “stealth” effect and offer an additional tumor targeting mechanism based on enhanced permeability and retention (EPR) effect^16^. Finally, owing to good absorption of light in visible and near-infrared region, covering the window of relative biological transparency (650–900 nm), B NPs can serve as contrast agents in photoacoustic imaging^17^ and sensitizers of photothermal therapy^17,18^. However, the fabrication of B-based nanoformulations suitable for biomedical use presents a real challenge^19^. As an example, chemical synthesis pathways are typically complicated and cause the contamination of NPs by toxic reaction products, such as carbon, chlorine, etc., while the size of formed NFs often exceeds several hundreds of nm^17,18,20,21^. On the other hand, dry fabrication methods such as laser pyrolysis do not render possible fine control of size, crystallinity and aggregation characteristics, while NPs prepared by these methods cannot easily be water-dispersed^22–24^.

Based on the natural production of nanoclusters under the action of laser radiation on a solid target, followed by their release into liquid ambient^25–28^, pulsed laser ablation in liquids (PLAL) has recently appeared as a novel “physical” nanofabrication route, which promises the synthesis of a variety of novel unique NFs^29,30^. As one of advantages, PLAL can be performed in ultrapure environment (deionized water, some organics) to exclude any contamination of the NPs surface^28^, while solutions of NPs prepared by laser ablation can be stable even without ligand protection due to electrostatic repulsion effect^31^. Another advantage is that the oxidation of formed nanomaterials can be minimized by decreasing the content of oxygen in water^32^ or the employment of organic solutions such as acetone^26^. Ultrashort laser ablation (femtosecond, nanosecond) showed the best efficiency among PLAL approaches in terms of control of size and stability characteristics of formed NPs, as well as the fabrication of complex nanoarchitectures^28,31^. In particular, we previously showed that techniques of femtosecond laser ablation and fragmentation can be used to fabricate stable solutions of low-size dispersed NPs of a variety of materials, including Au^28,31^, Si^32,34^, TiN^33,35^, elemental Bi^36^ NPs. Recently, methods of laser ablation from a Fe-B alloy target were elaborated for the fabrication of composite Fe-B NPs^37^. The experiments were carried out in acetone in the presence of ligands (polyvinyl pyrrolidone, PVP) to control size characteristics and minimize oxidation effects, which could alter material stoichiometry. The formed NPs were considered as a promising object for combined neutron boron capture therapy and magnetic resonance imaging modalities. Despite promising characteristics, the formed NPs could have been contaminated by products of acetone decomposition (typically carbon-based compounds) due to high energy of nanosecond laser radiation used in these experiments, as it was earlier reported^38–40^, while the concentration of boron in alloy nanostructures was still not very high, limiting further prospects in BNCT.

Here, we describe a laser-ablative methodology to fabricate stable aqueous solutions of pure elemental boron NPs, which should provide maximal concentration of boron atoms for BNCT and avoid any toxic contamination, as recommended by current state-of-the-art. In this methodology, we do not use organics to minimize oxidation effect. Instead, we perform fs laser ablation in deionized water in the absence of ligands, which excludes any carbon contamination of NPs. We show that laser synthesis process leads to the formation of aqueous solutions of elemental crystalline boron NPs with the averaged modal size of about 37 nm and boric acid (H_3_BO_3_), which can then be easily removed from the aqueous solution. We then describe a protocol for the coating of laser-synthesized B NPs with PEG and assess the cytotoxicity of formed NPs in vitro. We believe that synthesized B NPs can be very promising for biomedical applications.

## Results

### Synthesis and characterization of NPs

**Figure 1:**
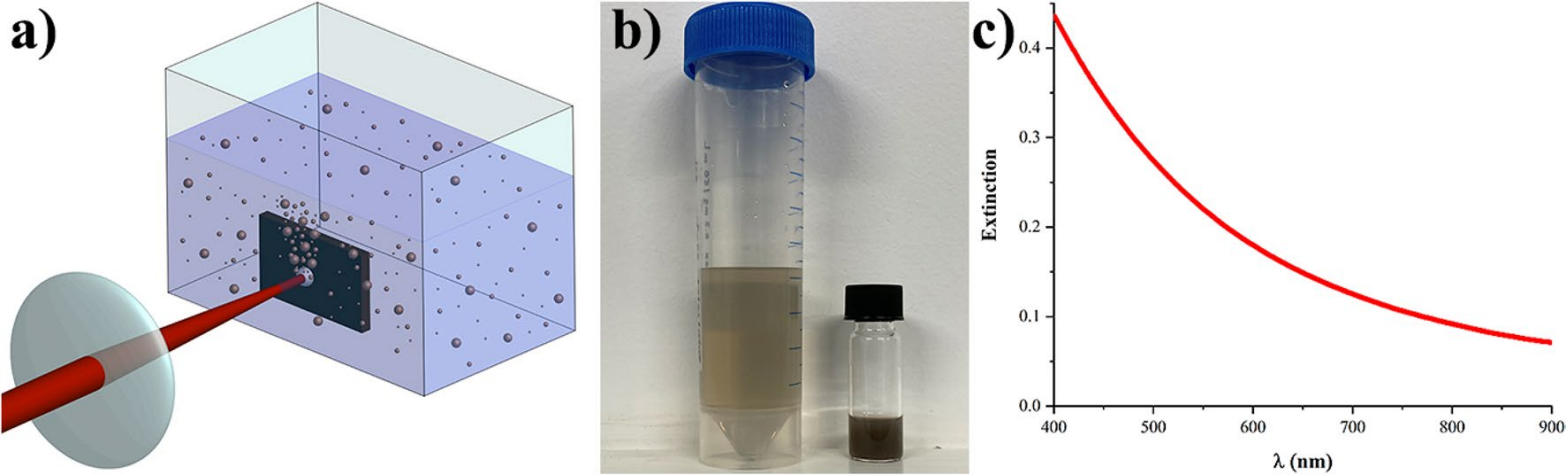
Boron NPs produced by laser ablation in deionized water. (**a**) Schematic of laser ablation experiment. (**b**) Initial (left) and concentrated (right) solutions of laser-ablated B NPs in deionized water. (**c**) Extinction spectrum from a colloidal solution of B NPs.

We performed laser ablation from elemental boron (B) bulk target in aqueous solutions according to a protocol described in Methods section. Briefly, the target was fixed vertically in a quartz cuvette and filled with deionized water or in water bubbled with argon (Ar) gas to pump out dissolved oxygen (Fig. 1a). A beam from a femtosecond laser was directed through one of the walls of the cuvette and focused by a converging lens on the target surface, while the cuvette was constantly moved by the translation stage to avoid ablation from the same area. We observed a brownish coloration of the solution after 30 minutes of the irradiation onset, which could be attributed to the formation of NPs. Final solutions were brown and transparent for as-prepared solutions and dark brown for concentrated ones, as shown in Fig. 1b, while their prolonged aging at room temperature did not reveal any sign of material precipitation suggesting a high colloidal stability. As shown in Fig. 1c, optical extinction spectra recorded using UV/Vis spectrometer did not contain any particular features, but has a long tail extending from visible to near-infrared region. It is important that the extinction efficiency in this tail was relatively high in the window of relative biological transparency (650–900 nm), which is in agreement with previously reported optical extinction data for elemental boron NPs^17^, suggesting the possibility of NPs applications in photoacoustic imaging photothermal therapy.

**Figure 2:**
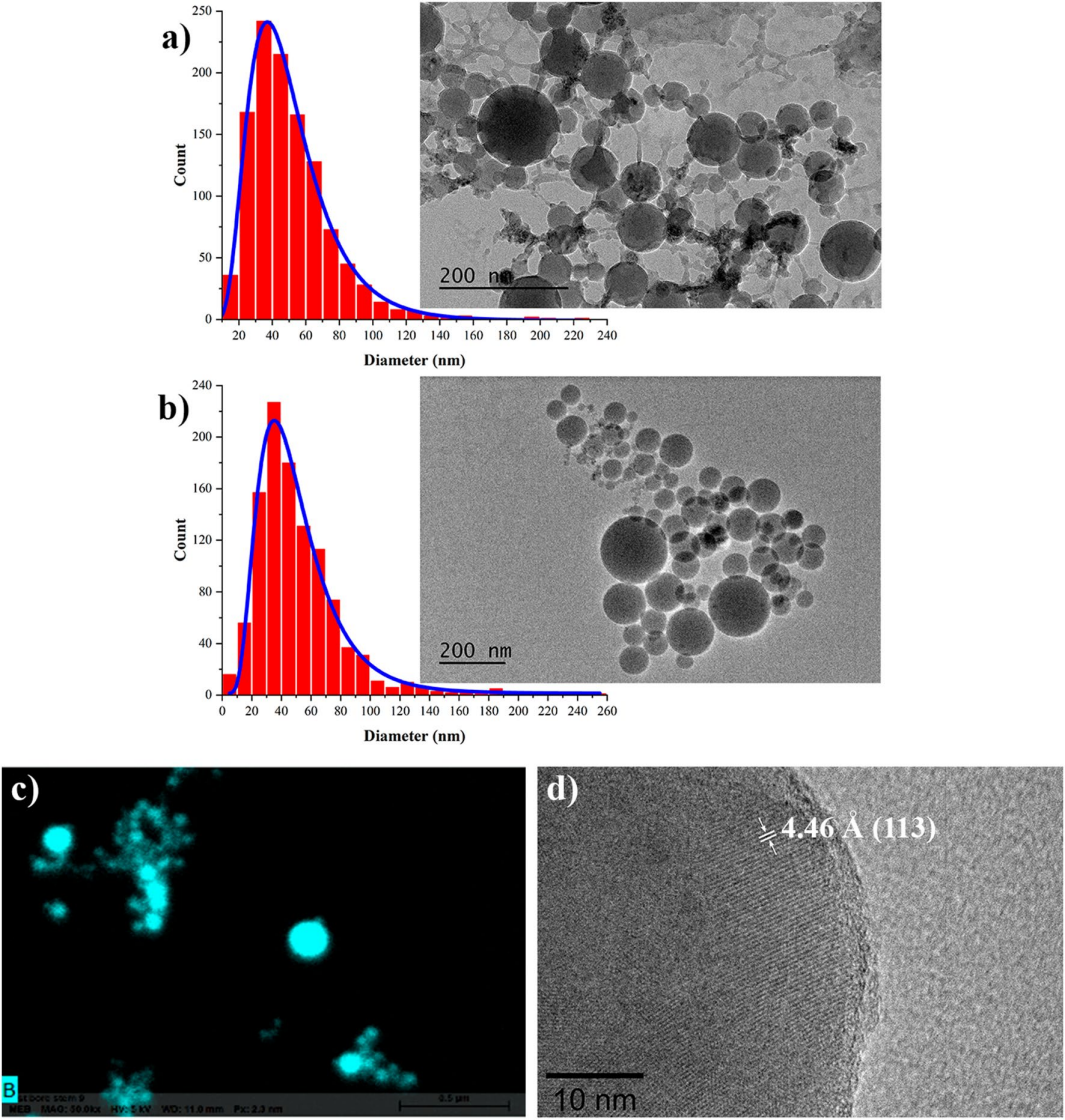
High resolution electron microscopy images of laser-synthesized NPs: (**a**) Typical TEM image (inset) and corresponding size distribution of as-prepared boron-based NPs. (**b**) Typical TEM image (inset) and corresponding size distribution of boron NPs after multiple centrifugation and purification steps. (**c**) Typical EDX map of NPs prepared by fs laser ablation in water. Cyan color corresponds to boron. (**d**) HR-TEM image of a polycrystalline nanostructure.

Our tests using transmission electron microscopy (TEM) showed that the prepared solutions were mainly composed of spherical NPs (inset in Fig. 2a), while their averaged (mode) diameter was 37 nm (Fig. 2a), as followed from our statistical analysis. According to Energy-dispersive X-ray spectroscopy (EDX) analysis, the formed nanostructures contained a significant amount of boron detected in regions where high contrast NPs were located (Fig. 2c), suggesting the domination of this element in the nanoparticle composition. In addition, examining TEM images, we could observe a lower contrast shell coating of NPs and elongated interconnections between the adjacent NPs (Fig. 2a). The presence of such low-contrast structures is consistent with the formation of boron-related compounds (boric acid, boron oxide) crystallized on the surface after drying of NPs on a TEM grid. To clarify the origin of these structures, we separated the NPs from the solution by centrifugation with subsequent drying of the latter, which resulted in the formation of a white crystalline solid powder. The resulting powder was examined by X-ray diffraction (XRD) spectroscopy and these tests revealed XRD peaks corresponding to boric acid H_3_BO_3_ (reference ICDD 01-073-2158), suggesting its formation during laser synthesis as a by-product (Fig. S1 from Supplementary Information). The examination of dried NPs-based powder also revealed the predominance of XRD peaks associated with boric acid, although other much weaker peaks related to boron, its oxide states and contaminations were also detected (spectrum not shown). We suppose that boric acid can be formed as a by-product according to the following pathways. As a first scenario, laser ablation of boron in highly oxidizing water ambient can result in the formation of boron oxide B_2_O_3_, while moderate water-solubility of B_2_O_3_ can lead to its hydration to produce H_3_BO_3_. As another possibility, it could be direct laser-ablative formation of B^3+^ ions and dissociative electrons to cause the decomposition of water molecules into ions (OH^−^), which then react with boron to provide boric acid production similarly to how it was reported previously for other materials^41^.

**Figure 3:**
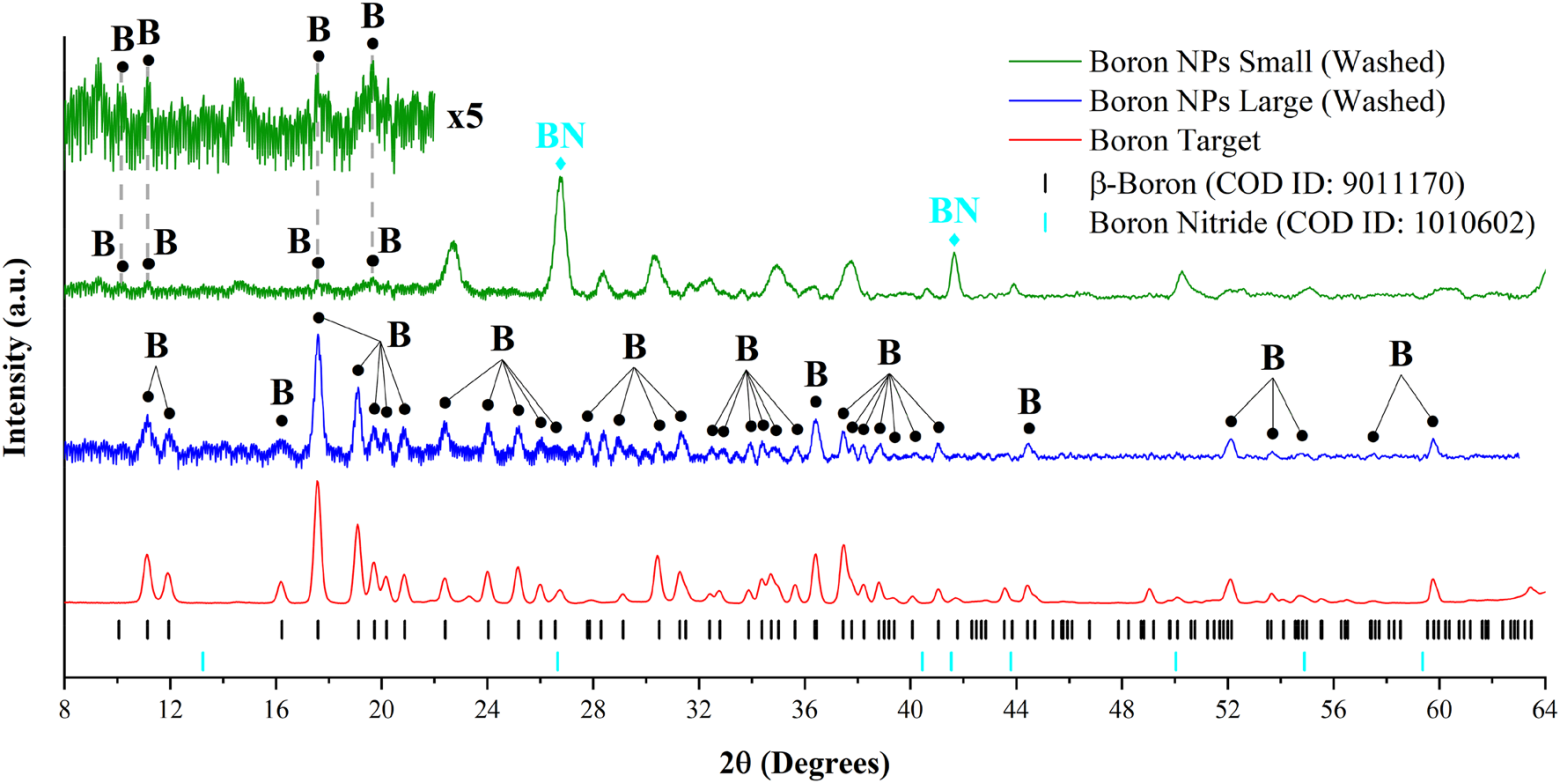
X-ray diffraction **(**XRD) measurements of NPs produced by laser ablation in deionized water after centrifugation and purification steps. XRD spectra for large and small size populations of NPs are shown by blue and green lines, respectively. The inset shows a magnified XRD spectrum for most intense lines of B. Lower lines show XRD patterns for crystalline boron (black) and boron nitride (cyan).

Identifying boric acid as an important by-product of synthesis, we applied a purification procedure based on multiple centrifugation steps in order to extract the remaining boron-based nanoformulations in pure state. Then, we washed them with deionized water to remove the excess of the boric acid residues. A typical TEM image of boron NPs after the purification step is shown in Fig. 2b. One can see that the NPs were ideally spherical, while the characteristic low-contrast shell coatings and interconnections between adjacent NPs were absent, suggesting that these structures were indeed related to boric acid and then washed out during the purification. As shown in Fig. 2b, the averaged size of NPs (~ 40 nm) was nearly identical to the size of NPs before purification. It should be noted that in addition to relatively small NPs (tens of nm) forming the majority of nanoparticle population, we observed a certain number of larger NPs (several hundreds of nm). Since we mainly focused our attention on the fabrication of small NPs (tens of nm), which have better prospects for biomedical applications, larger NPs were separated by a centrifugation step. Nevertheless, we examined both large and small size NPs populations by XRD. As shown in Fig. 3 (blue line), large size population provided XRD spectrum corresponding to crystalline boron phase (reference ICDD 00-031-0207), which nearly matched the spectrum of used boron target (red line). Although we may not exclude size-dependent crystallinity for laser-ablated NPs, we believe that the observed signals were generated by large fragments detached from the hot-pressed target during the ablation process. In contrast, XRD spectra from small NPs fraction (Fig. 3, green line) were characterized by a drastic loss of intensity of boron-related peaks, as we could observe only very weak signals from most intense XRD signatures. We conclude that NPs from small size population are mainly composed of amorphous boron, although the crystalline phase is also present to some extent, but is not well resolvable by the XRD technique. It should be noted that we recorded several additional strong peaks in the XRD spectra of small NPs, which could be attributed to boron nitride (BN) and residuals of boric acid. We believe that the appearance of BN features is an artifact related to catalytic activity of boron NPs under partial nitrogen exposure during their preparation of samples in atmospheric air^42,43^. The supposition of certain extent of crystallinity of small boron NPs was confirmed by the analysis of high-resolution TEM (HR-TEM) images. As shown in Fig. 2d polycrystalline structure is clearly resolvable despite a significant presence of the amorphous phase. The calculated interplanar spacing is 4.46 Å, which corresponds to the (113) plane of the elemental β-boron, suggesting the presence of a substantial fraction of crystalline boron in the nanoparticle composition.

To clarify the ratio of boron and boron acid in the outcome of laser-ablative synthesis, solutions of NPs were examined using inductively coupled plasma mass spectrometry (ICP-MS). These measurements confirmed that boric acid was the dominating component, but elemental boron NPs was also detected. As follows from ICP-MS data, boric acid contained about 2 times more boron than NPs *n*_*boron*_*/n*_*boric acid*_ = 0.54.

**Figure 4:**
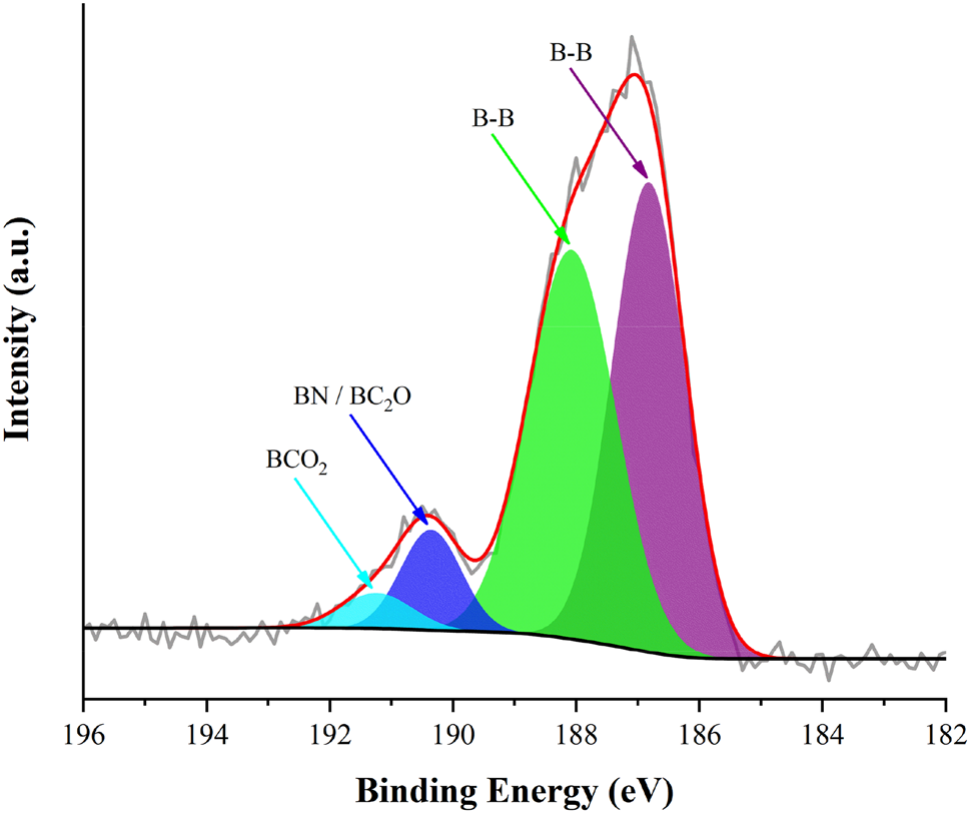
X-ray photoelectron spectroscopy (XPS) measurements in B 1 s region of washed B NPs produced without Ar bubbling.

To clarify the composition of the upper layer of laser-synthesized NPs, we performed X-ray photoelectron spectroscopy (XPS) measurements of purified samples in B 1 s region, which provide information on the composition of the upper surface layer of NPs. Results of the analysis are shown in Fig. 4. One can see the appearance of peaks at 186.8 and 188.1 eV, which are typically attributed to B-B bonds to confirm the presence of elemental boron^44–47^. Interestingly, the deconvolution of the spectra reveals the appearance of two additional peaks at 190.4 and 191.2 eV, evidencing the formation of boron nitride-based^48^ and boron carbide-based (boronic acid groups)^49–51^ compounds on the nanoparticle surface. The production of carbide-based residues can be due to contamination of the boron target with carbon, whereas the formation of boron nitride was unexpected. The XPS survey of such samples (Fig. S2a) confirms the presence of a peak in N 1 s region. We believe that the formation of nitrogen-related compounds on the surface of NPs can be explained by the exposure of samples to ambient air during sample preparation and drying steps, which led to catalytic reaction with nitrogen.

Taking into account a major role of the oxidation phenomena in the formation of boron NPs and boron-containing substances (boric acid), we carried out comparative laser-ablative experiments in water bubbled with argon, before and during the experiment (degassed water) to pump out dissolved oxygen, similarly to how it was done with Si nanoparticles in our previous paper^32^. Our tests showed that this case is also characterized by the formation of boric acid, as confirmed by XRD and ICP-MS data (not shown), but the ablation in degassed water provided a higher proportion of boron *n*_*boron*_*/n*_*boric acid*_ = 2.45, as reported by ICP-MS. However, the difference in structural properties of boron NPs prepared by both methods was not substantial, as it took place in the case of Si nanoparticles^32^. Indeed, TEM analysis did not reveal any significant deviation of the averaged diameter of NPs prepared in degassed and non-degassed samples (Figs. S3 and 2b, respectively) while XRD (not shown for degassed NPs) and XPS (Figs. S4 and 4, respectively) evidenced similar properties of NPs prepared by both methods. It should be noted that similarly to the non-degassed case, we recorded boron nitride-related peak in XPS spectrum of NPs prepared in Ar-bubbled ambient (Figs. S4 and S2b). This fact supports our supposition on the appearance of this signal as a result of catalytic activity of boron during post-fabrication exposition of samples to air. Indeed, since Ar-bubbled water is free of nitrogen, there is no N_2_ source to produce BN compounds. As a conclusion from these tests, the minimization of oxygen content led to the increase of B NPs content, but did not change structural properties of formed NPs.

Our experiments showed that after re-dispersion in deionized water purified Boron NPs rapidly agglomerated and precipitated in a form of turbid brown flakes, while as-prepared solutions were stable for months of storage at ambient conditions. A relatively high stability of colloidal NPs solutions prepared by laser ablation in liquids is generally attributed to their electrostatic stabilization as a result of NPs charging. The origin of the NPs electrical charge is not well understood, but it is widely attributed to oxidation of the ablated material in high temperature conditions during early stages after laser-target interactions or to adsorption of charged molecules from the liquid. The latter mechanism generally requires addition of some substances to liquid, which will be absorbed on the NPs surface. However, it seems that boric acid, appearing as a by-product of synthesis during laser ablation, serves as a stabilizing agent for the formed boron NPs. We believe that this by-product adsorbs on the NPs surface and forms an electrical double layer, which stabilizes the NPs solution. This hypothesis is supported by the fact that ζ–potential of NPs increased from − 36 mV in the as-prepared colloid to almost neutral value in the purified colloid, additionally indicating the loss of stability after the multiple purification steps.

### Functionalization and biological assessment of laser-synthesized boron NPs

**Figure 5:**
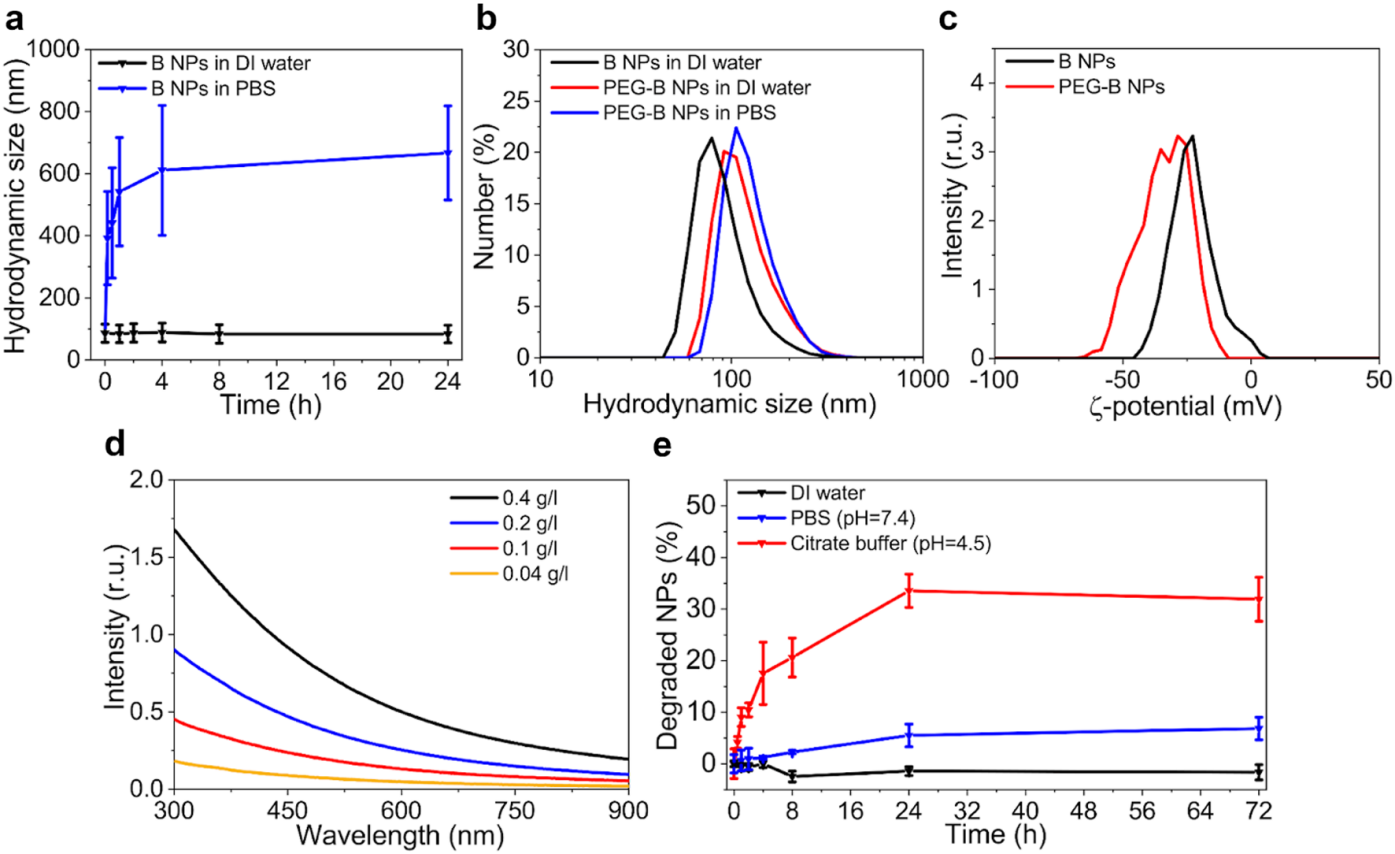
Colloidal stability tests: (**a**) The evolution of hydrodynamic size of boron NPs incubated in distilled water and PBS. (**b**) Hydrodynamic sizes of boron and PEG-B NPs in water and PBS. (**c**) ζ-potential distributions of boron and PEG-B NPs. (**d**) Extinction spectra of boron NPs at 0.04–0.4 g/l concentrations in distilled water. (**e**) Kinetics of boron NP degradation in water, PBS (pH = 7.4) and citrate buffer (pH = 4.5).

As follows from our analysis, the purification of NPs from boric acid residuals and re-dispersion in water led to the loss of colloidal NPs stability. The mean hydrodynamic size of NPs was much larger (92 nm) and did not show any changes within 1 day, whereas their incubation in phosphate buffered saline (PBS) rapidly led to a significant increase of the mean particle size, started to reach the value of 600 nm within 4 h (Fig. 5a). This indicates the colloidal instability of boron NPs due to aggregation in the buffers.

To prevent nanoparticle interaction, we coated them with polyethylene glycol containing carboxyl terminate groups. For this aim, we used a reaction of silane-PEG hydrolysis and condensation of derivatives on the surface of boron particles in alkaline conditions. After the coating, the hydrodynamic diameter of boron NPs in distilled water slightly increased from 92 ± 34 to 121 ± 45 nm, which confirmed the attachment of the polymer to the particle core (Fig. 5b). In addition, we observed the increase of particle colloidal stability in PBS with no signs of any aggregation (Fig. 5b). Hydrodynamic size of B-PEG particles in PBS was 128 ± 42 nm. In addition, we observed a shift of ζ-potential of boron NPs after PEGylation to more negative values, which indicated the increase of repulsion between particles (Fig. 5c). Thus, silane based PEGylation could be applied for boron NPs stabilization in physiologically relevant conditions.

To study the degradability of boron NPs in buffer solutions we applied a spectrophotometry-based approach. The extinction spectra of boron NPs (Fig. 5d) made possible a quantitative determination of particle concentration in aqueous solutions (see calibration curve at Fig. S5). It could be seen in Fig. 5e, that 5% of boron NPs (0.1 g/l) degrade in a PBS (pH = 7.4) within 3 days. A rapid and significant 32% decrease in particle concentration was found in acidic citrate buffer (pH = 4.5) after 1 day, which indicates the ability of boron NPs to be partly dissolved in the lysosomal conditions.

**Figure 6:**
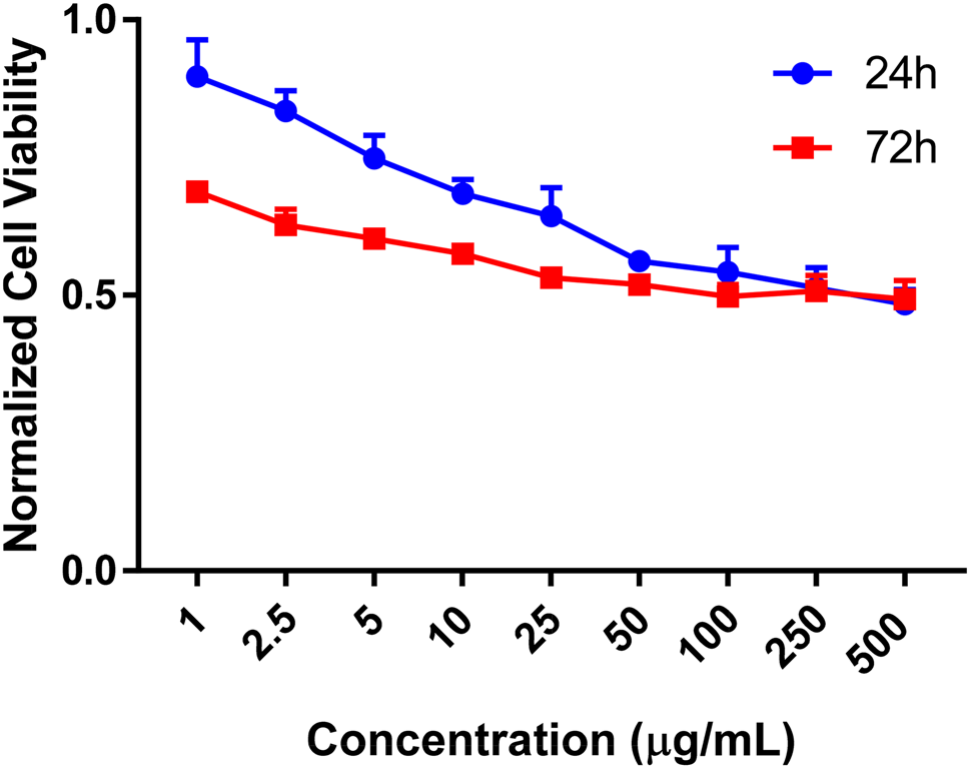
Cytotoxicity evaluation of boron NPs.

The cytotoxicity of B NPs was evaluated in HeLa cells using a standard MTT assay which measures the reduction of 3-(4,5-Dimethylthiazol-2-yl)-2,5-diphenyltetrazolium bromide into an insoluble formazan product. This process occurs in the mitochondria of live cells as a result of cellular respiration. B NPs showed slightly less toxicity at concentrations between 1 and 100 µg/mL at 24 h than at 72 h (Figure 6). The IC_50_ cannot be determined from this data, but at both 24 and 72 h even when treated with concentrations of 500 µg/mL only about 50% cell death was observed. This data suggests bare B NPs are relatively well tolerated by cells.

## Discussion

Thus, our experiments clearly demonstrated the possibility of laser synthesis of spherical boron-based NPs in organic-free environment, which present a promising object for biomedical and other applications. Our characterization tests showed that these NPs are composed of elemental boron. Initially generated with a significant amount of boric oxide as an oxidation by-product of laser synthesis, boron NPs were later purified from boric acid by an easy centrifugation step.

One of interesting conclusions from obtained experimental data is related to unusual physico-chemical conditions of laser synthesis, which led to the formation of elemental boron NPs. The ablation was performed in aqueous ambience, which is well compatible with biological systems and ideal for the synthesis of nanomaterials for biomedical applications. On the other hand, presenting a highly oxidizing medium, water causes a strong oxidation of most materials, which can dramatically alter their properties. As an example, even in the case of chemically inert gold NPs the oxidation phenomena lead to the formation a slight gold oxide shell, terminated by O^−^ group under pH > 5^31^, opening avenues for its unusual interactions, via hydrogen bonding, with OH groups of important polymers such as cyclodextrins^52^, dextran^53^, polyethylene glycol^53^, proteins^54^ and oligonucleotides^55^. and rendering possible their one-step functionalization. For other materials the oxidation phenomena are even more significant, while their contribution depends on physico-chemical characteristics of materials. Several strategies to avoid or minimize oxidation phenomena were proposed. In the case of weakly oxidizing materials such as silicon (Si), a strategy based on pumping out oxygen dissolved in water by its bubbling with an inert gas (e.g., argon)^32^ is sufficient to exclude oxidation during a fast stage of NP core synthesis, while the upper thin oxide shell is formed during a further aging of Si NPs in water ambience^56^. In the case of stronger oxidizing materials, another strategy based on laser ablation in organic solvents (ethanol, acetone, hexane, etc.) is used, which makes possible a drastic decrease of oxidation effects. In particular, this strategy was used in our earlier works on the laser synthesis of TiN^33,35^ and Bi^36^ NPs. Torresan et al^37^ also used the latter straightforward strategy for the fabrication of Fe-B NPs, while polyvinyl pyrrolidone (PVP) was used to control size characteristics. However, the used highly energetic ns laser radiation could lead to a partial decomposition of acetone, as it was observed in earlier studies^38,39^, which can lead to a partial contamination of NPs, while formed NPs required further transfer to water. Our methodology did not follow the currently existing paradigm to avoid oxidation of formed material during laser-ablative synthesis. Indeed, instead of performing ablation experiments in low-oxidizing organic environment, we did it in strongly oxidizing deionized water (without pumping out dissolved oxygen). Surprisingly, the laser-ablative process followed unusual scenario leading to the formation of elemental boron NPs together with boric acid (H_3_BO_3_) as an oxidation by-product. Furthermore, boric acid played an important role as it led to the stabilization of colloidal aqueous solutions of boron NPs. It is important that B NPs could later been purified from boric acid by a simple purification step and then coated by PEG to stabilize aqueous colloidal solutions of NPs and improve their biocompatibility. We believe that such strategy requires further detailed investigation and can be applied for the synthesis of a variety of other nanomaterials.

It is important that the NPs demonstrated a low cytotoxicity profile in MTT tests, which promises their successful use in biological systems. We believe that the synthesis of NPs of pure elemental boron is an important advance in theranostic (therapy + diagnostics) modalities based on boron-based compounds. First, a strong absorption value of these NPs in the first window of biological transparency promises the employment of such NPs as sensitizers of phototherapy. In such a modality, PEG-coated B NPs will be delivered to tumor region via active targeting (see, e.g., Ref^57^) or passive accumulation due to EPR effect^16^ and then heated by photons to provide local overheating (hyperthermia) of adjacent cancer cells in order to initiate their death, similarly to how it takes place in cases of laser-synthesized TiN^33^ and Si^58^ NPs. The presence of a strong absorption also means the possibility of enabling imaging modalities based on photoacoustic tomography^17^. Finally, the synthesized B NPs look very appealing for applications in boron neutron capture therapy (BNCT)^5–8^. Since the formed NPs are composed of elemental boron, they should provide the highest possible concentration of boron atoms in tumor cells to maximize the therapeutic outcome. It should be noted that in our experiments we used targets of naturally abundant boron, which contains about 20% of neutron-active ^10^B isotope. We suppose that the proposed laser-ablative methodology should work in the case of ^10^B-enriched targets to synthesize NPs of elemental ^10^B isotope.

## Conclusion

We demonstrated the synthesis of pure boron NPs by femtosecond laser ablation in deionized water. We show that laser synthesis process leads to the formation of elemental boron NPs with the averaged size of about 40 nm, together with boric acid (H_3_BO_3_) as an oxidation by-product, which helps to stabilize the elemental boron NPs dispersion. We then demonstrate the possibility of purification of boron NPs from boric acid residuals via a series of centrifugation and washing steps and describe a protocol for the coating of NPs in order to improve solution stability and biocompatibility. Our tests in vitro illustrated low toxicity of laser-synthesized boron compounds. It was also found that the NPs exhibit relatively strong absorption over a broad spectral range, in the NIR window of relative tissue transparency, promising their use as contrast agents for photoacoustic imaging and sensitizers of phototherapy. The proposed strategy for the fabrication of elemental metal NPs in highly oxidizing water environment, followed by the exclusion of oxidized component (H_3_BO_3_ in our case) looks like a novel paradigm in the laser-ablative synthesis, which can be extended to other promising nanomaterials. We believe that synthesized B NPs can be very promising for biomedical applications, including boron neutron capture therapy (BNCT) and phototherapy.

## Methods

### Synthesis of nanoparticles

Nanoparticles were synthesized by laser ablation of a hot-pressed boron target (MaTecK Material Technologie & Kristalle GmbH, Juelich, Germany, purity 99.5%, natural isotopic composition) in deionized water (18.2 MΩ at 25 °C) at ambient conditions or in water bubbled with argon (Ar) gas to pump out dissolved oxygen. A beam coming from a Yb:KGW laser (1025 nm, 480 fs, 8 kHz, Amplitude Systems, France or 1030 nm, 270 fs, Avesta, TETA 10 model, Moscow, Russia) was focused on the surface of the boron target fixed vertically in a quartz cuvette filled with 50 mL of deionized water. The energy was preliminary attenuated to the value of 350 µJ per pulse using half-wave plate and Bruster polarizer. The liquid layer thickness between the inner wall of the cuvette and the surface of the target was 3.3 mm. Due to the high energy of the interacting beam, self-focusing effects take place, therefore, the quartz cuvette was moved forward in the direction of the convex lens to focus the beam on the surface of the target and to avoid damaging of the glass wall. To prevent the ablation from the same region, the cuvette with the target was constantly moved by a translation stage. The scan area was set to 7 × 7 mm and the displacement speed to 2.5 mm s^−1^. The laser ablation in water lasted for 7 h.

### Nanoparticles characterization

Boron nanoparticles size, shape and composition were characterized using transmission electron microscopy (TEM, JEOL JEM-3010, 250 kV acceleration voltage, W filament and JEOL JEM-2100F, 200 kV, ZrO/W FEG) and scanning electron microscopy (SEM, JEOL JSM-7900F, 0…30 kV) equipped with EDX detector (Brüker). For that, 10 µL of elemental boron particles solution was dropped on the copper-carbon grid (200 mesh, Oxford Instrument) and dried overnight under ambient conditions. Size distribution, fast Fourier transform (FFT) and inverse fast Fourier transform (IFFT) analysis were performed in Fiji ImageJ software. Extinction spectrum was recorded using a UV–Visible spectrophotometer (UV-2600, Shimadzu) and a rectangular quartz cuvette with an optical pathlength of 10 mm.

XRD measurements of dried elemental B NPs powder were performed in a transmission mode. The instrument is equipped with a double reflection mirror (Osmic), an image plate detector (Mar345), and a high brilliancy rotating anode (Rigaku RU-200BH, 50 kV, 50 mA). The radiation is Cu Kα (λ = 1.5418 Å), and the size of the beam is 0.5 × 0.5 mm^2^. The maximum 2θ value is 65° (0.3° experimental resolution). The powder was obtained by drying a concentrated solution of B NPs in deionized water under ambient conditions. The XRD reflexes were calculated using VESTA (Visualization for Electronic and STructural Analysis) software^59^ and the data files from Crystallography Open Database (COD ID files: 9011170 for β-Boron, 1010602 for boron nitride and 9014010 for Boric Acid)^60,61^.

The XPS spectra were recorded using Physical Electronic PHI Versaprobe 5000 equipped with a hemispherical energy analyzer. The hemispherical analyzer was operated in Fixed Analyzer Transmission (FAT) Mode. A monochromic Al Ka X-ray source (1486.60 eV) was operated at 25 W and 15 kV with a beam diameter of 100.0 µm. The energy of the analyzer was operated at a pass energy of 117.5 eV for survey acquisitions and 23.50 eV for high-resolution acquisitions. The energy resolution was 0.020 eV for high resolution spectra, and 1.0 eV for survey spectra. The operating pressure of XPS was around 3.5 × 10^−6^ Pa. Dual charge neutralization was utilized to reduce the effects of charging on the acquired signal. Survey acquisitions were taken for binding energies from 0 to 1000 eV. To prepare the samples for the XPS measurements, washed B NPs in water were dropped on a gold-coated (210 nm thickness) silicon substrate and dried overnight under ambient conditions. The spectrum background was fitted using Shirley correction.

Boron concentration in nanoparticles and supernatant were measured using inductively coupled plasma mass spectrometry (ICP-MS). Nanoparticles were centrifuged at 15000 g, 30 min, supernatant and particle residue were separated and dissolved in 10% nitric acid under the heating at 80 °C. A mass-spectrometer (NexION 2000, PerkinElmer, USA) was calibrated before the analysis using serial dilutions of sodium tetraborate (Sigma-Aldrich, USA) with known concentrations. ^11^B peak was used for analysis.

### Nanoparticles purification

To purify B NPs from the excess of boric acid formed during laser ablation, centrifugation was performed. First, the solution was centrifuged at 5000 g 5 min to separate large particles. Then, the solution was centrifuged twice at 15000 g 15 min and redispersed in deionized water at each step.

### Nanoparticle coating

To increase boron nanoparticle stability in buffers they were coated with polyethylene glycol (PEG). For this aim 1 mg of particles were dissolved in 1 mL of 95% ethanol and mixed with 100 µL of ethanol containing 100 µg of Silane-PEG (5kDa) and 10 µg of Silane-PEG-carboxyl (5kDa). After that 10 µL of 30% ammonia hydroxide was added to accelerate hydrolysis of silanes. Solution of boron nanoparticles was heated at 60 °C for 2h and then washed 2 times from unreacted chemicals with water by centrifugation (5 000 g 5 min, then 15 000 g, 15 min).

### Functionalized nanoparticles characterization

The hydrodynamic diameter and ζ-potential measurements were performed by dynamic and electrophoretic light scattering, respectively, using a Zetasizer Nano ZS (Malvern Instruments, UK) device. Number weighted size distributions were used for analysis. ζ-potential measurements were performed in a 10 mM NaCl solution using the Smoluchowski approximation.

Extinction spectra of aqueous colloids of nanoparticles were recorded by an Infinite M1000 PRO spectrophotometer (Tecan, Austria).

### Degradation kinetics measurement

To study boron NP chemical stability in distilled water, PBS (pH = 7.4) or citrate buffer (pH = 4.5), 100 µg of particles were added to 1 ml of the corresponding medium and rigorously mixed. The extinction intensity of NPs at 500 nm was measured to analyze particle concentration at each time point. The obtained intensities were averaged (n = 3) and normalized to the initial signal to determine the content of undissolved particles. Before each measurement samples were thoroughly sonicated and stirred.

### Cells and culture conditions

HeLa cells (ATCC, Manassas, VA) were grown in 100 mm cell culture dishes and maintained in Advanced DMEM media, supplemented with 10% fetal bovine serum, 1% glutamax, 1% Antibiotic Antimycotic Solution at 37 °C in a humidified atmosphere containing 5% CO_2_.

### Cytotoxicity

Cytotoxicity of Boron NPs was determined by measuring the viability of cells by the standard MTT assay. Briefly, 2.5 × 10^3^ HeLa cells/well were plated in 96-well plates. After incubation for 24 h, 20 µl of the desired nanoparticle concentration was added to the well containing 180 µl of media. Untreated cells were used as a control. Following a 24 or 72 h incubation period media was removed, and cells were incubated with 100 µl 500 µg/mL methyl thiazolyl tetrazolium (MTT) for 2 h at 37 °C, 5% CO_2_. The MTT solution was removed, and the formazan reaction products were dissolved in DMSO overnight in the incubator. The optical density of the formazan solution was read on a microplate reader (BioTek Instruments, Inc., Winooski, VT) at 490 nm with a reference wavelength of 630 nm. Cell viability was normalized to untreated cells used as the control.

## Supplementary Information

Supplementary Information.
