# Supplementary material for: Laser-ablative aqueous synthesis and characterization of elemental boron nanoparticles for biomedical applications
Source: Sci Rep. 2022 Jun 1;12:9129. doi: 10.1038/s41598-022-13066-8 (PMC9159993; doi:10.1038/s41598-022-13066-8)
Supplement: Supplementary file 1 — Supplementary Information. [file 41598_2022_13066_MOESM1_ESM.docx]

**Supplementary Information**

**Laser-Ablative Aqueous Synthesis and Characterization of Elemental Boron Nanoparticles for Biomedical Applications**

# Andrei I. Pastukhov^1^, Iaroslav B. Belyaev^2,3^, Julia C. Bulmahn^4^, Ivan V. Zelepukin^2,3^, Anton A. Popov^2^, Irina N. Zavestovskaya^2,5^, Sergei M. Klimentov^2^, Sergey M. Deyev^2,3^, Paras N. Prasad^2,4^* and Andrei V. Kabashin^1^*

1Aix Marseille University, CNRS, LP3, Campus de Luminy, Case 917, 13288, Marseille, France

2MEPHI, Institute of Engineering Physics for Biomedicine (PhysBio), 115409 Moscow, Russia

3Shemyakin–Ovchinnikov Institute of Bioorganic Chemistry, Russian Academy of Sciences, 16/10 Miklukho-Maklaya St, Moscow, 117997, Russia

4University at Buffalo, The State University of New York, Department of Chemistry and the Institute for Lasers, Photonics, and Biophotonics, Buffalo, New York 14260, United States

5P. N. Lebedev Physical Institute of the Russian Acad. Sci., Leninskiy Pr. 53, 119991 Moscow, Russia

*[pnprasad@buffalo.edu](mailto:pnprasad@buffalo.edu), [andrei.kabashin@univ-amu.fr](mailto:andrei.kabashin@univ-amu.fr)


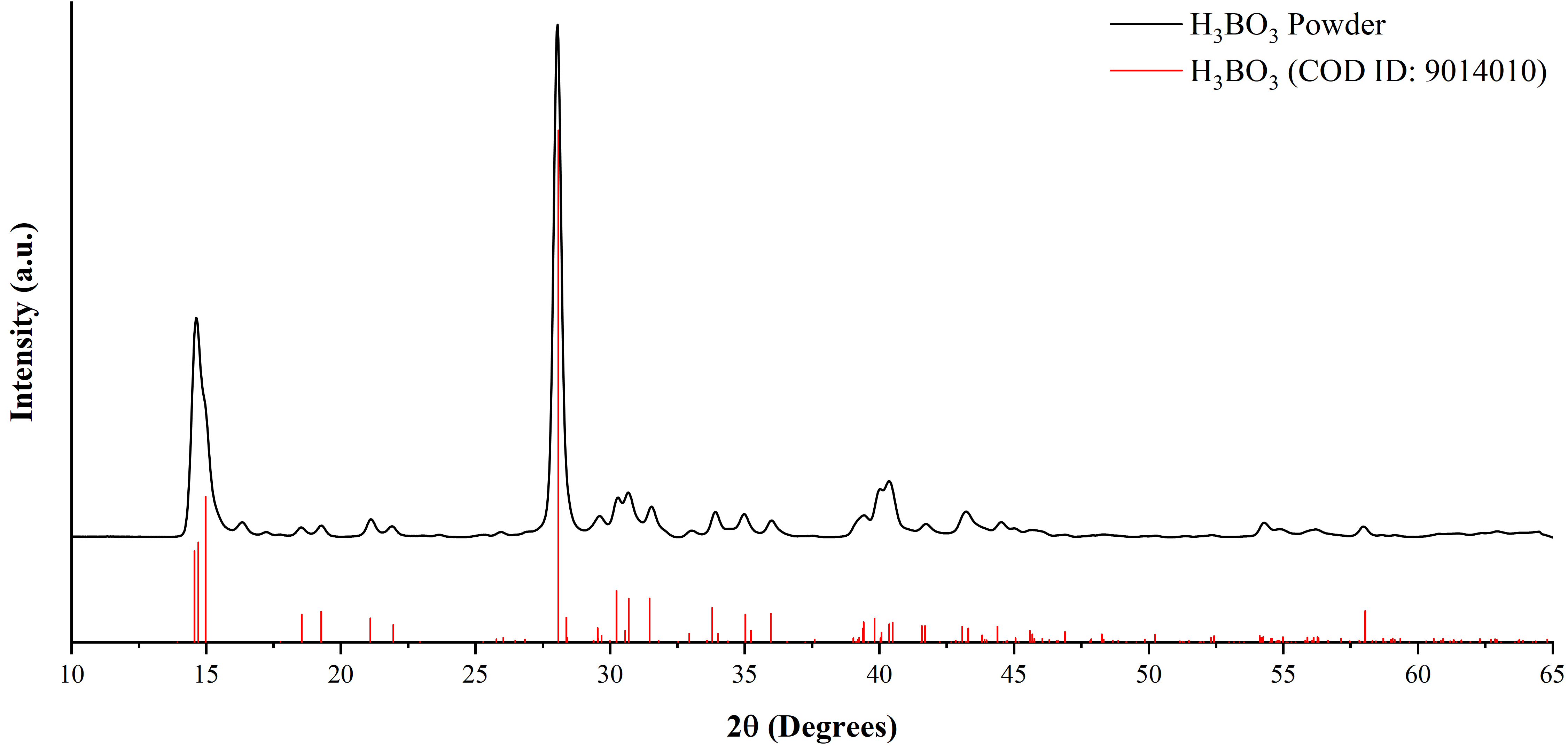


**Figure S1.** XRD pattern of dried boric acid powder from the solution.


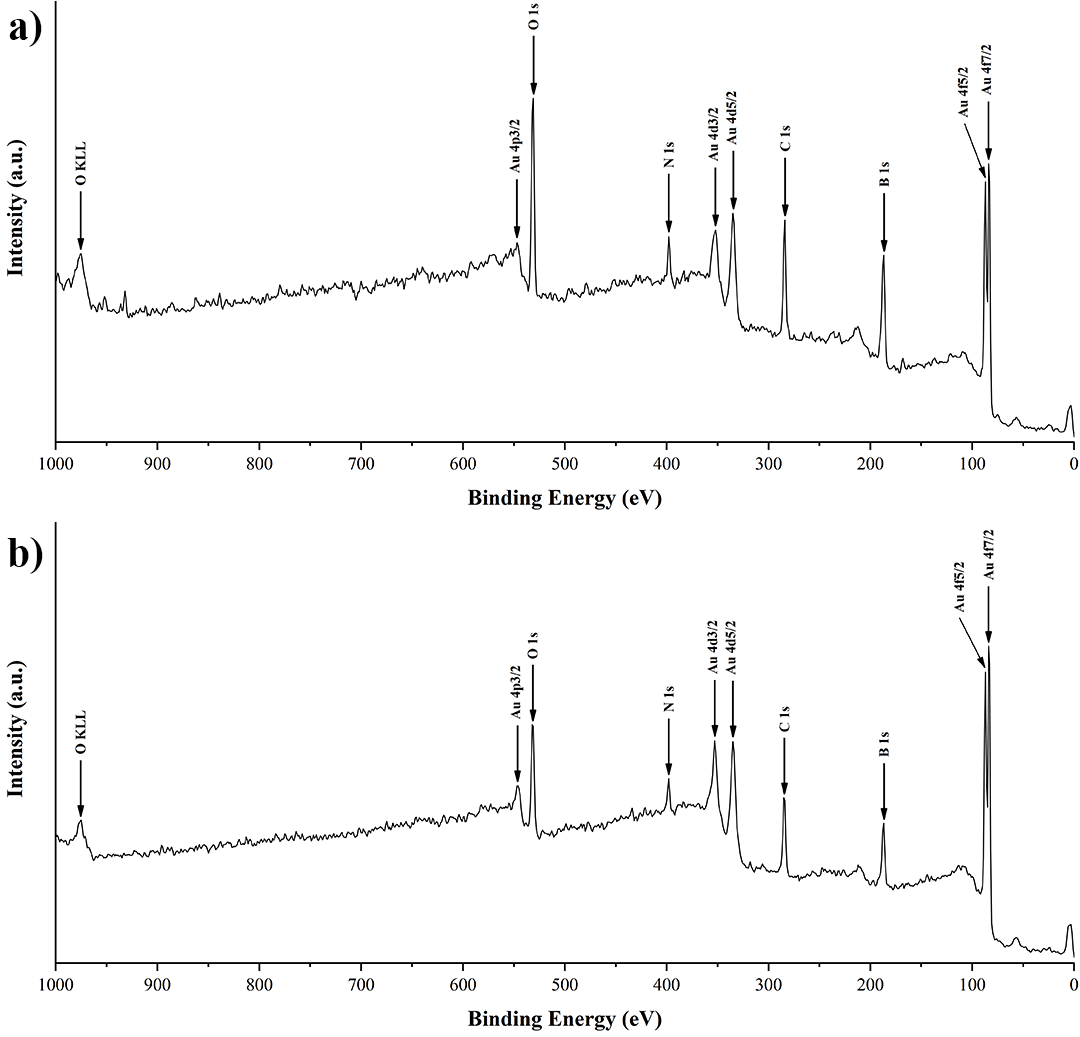


**Figure S2.** XPS survey spectra for samples of B NPs prepared by laser ablation in deionized water (non-degassed ambient) **(a)** and under bubbling of water with Ar gas (degassed ambient) **(b)**. Note that a strong Au signal comes from Si substrate covered with Au layer (210 nm).


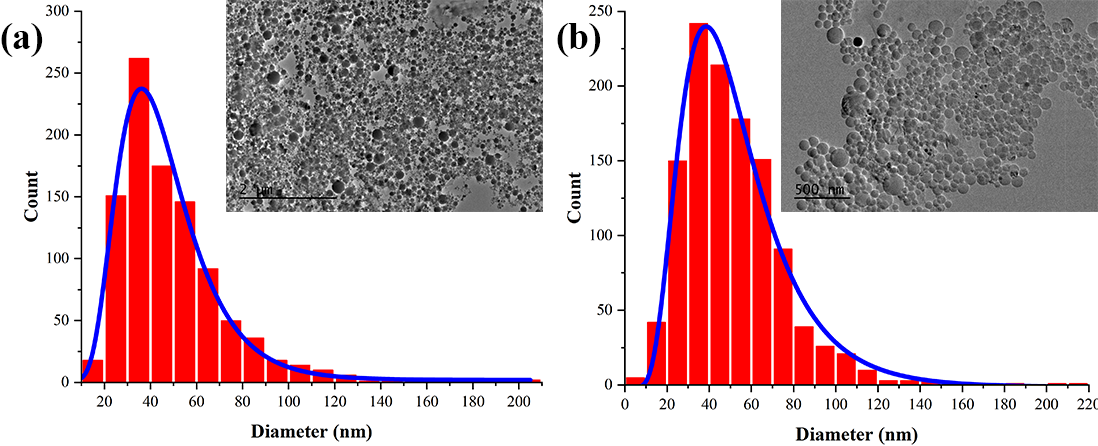


**Figure S3.** Size distribution analysis of boron-based NPs prepared by laser ablation from a B target in deionized water bubbled with Ar gas (degassed ambient): **(a)** As-prepared; **(b)** Purified.


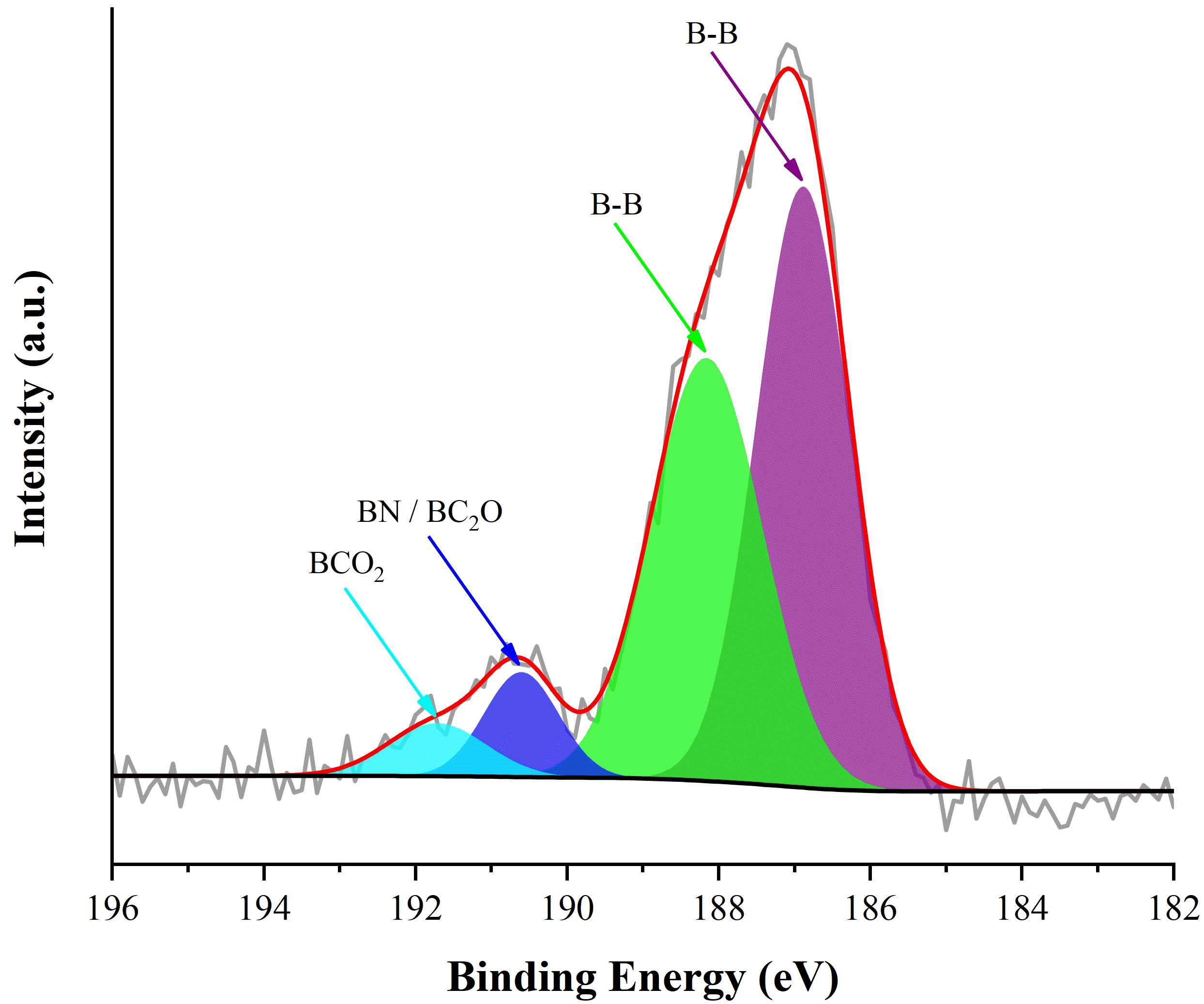


**Figure S4.** X-ray photoelectron spectroscopy (XPS) measurements in B 1s region of purified NPs prepared by laser ablation from a B target in deionized water bubbled with Ar gas


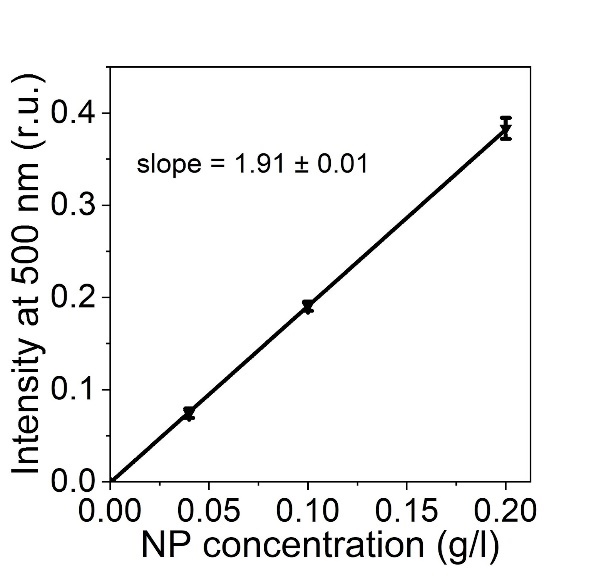


**Figure S5.** Calibration curve of the absorbance intensity of boron NPs at 500 nm in water versus concentration (n=3).
